# Extracellular vesicles as biomarkers for AIDS-associated non-Hodgkin lymphoma risk

**DOI:** 10.3389/fimmu.2023.1259007

**Published:** 2023-09-22

**Authors:** Laura E. Martínez, Larry I. Magpantay, Yu Guo, Priya Hegde, Roger Detels, Shehnaz K. Hussain, Marta Epeldegui

**Affiliations:** ^1^ UCLA AIDS Institute and David Geffen School of Medicine, University of California, Los Angeles, Los Angeles, CA, United States; ^2^ Department of Obstetrics and Gynecology, David Geffen School of Medicine, University of California, Los Angeles, Los Angeles, CA, United States; ^3^ Jonathan and Karin Fielding School of Public Health, University of California, Los Angeles, Los Angeles, CA, United States; ^4^ Department of Public Health Sciences, School of Medicine and Comprehensive Cancer Center, University of California, Davis, Davis, CA, United States; ^5^ Jonsson Comprehensive Cancer Center, University of California, Los Angeles, Los Angeles, CA, United States

**Keywords:** extracellular vesicles, biomarkers, AIDS-NHL, PD-L1, CD40, TNF-RII, IL-6Rα

## Abstract

**Introduction:**

Extracellular vesicles are membrane-bound structures secreted into the extracellular milieu by cells and can carry bioactive molecules. There is emerging evidence suggesting that EVs play a role in the diagnosis, treatment, and prognosis of certain cancers. In this study, we investigate the association of EVs bearing PD-L1 and molecules important in B-cell activation and differentiation with AIDS-NHL risk.

**Methods:**

EVs were isolated from archived serum collected prior to the diagnosis of AIDS-NHL in cases (N = 51) and matched HIV+ controls (N = 52) who were men enrolled in the Los Angeles site of the MACS/WIHS Combined Cohort Study (MWCCS). Serum specimens of AIDS-NHL cases were collected at a mean time of 1.25 years (range of 2 to 36 months) prior to an AIDS-NHL diagnosis. The expression of PD-L1 and other molecules on EVs (CD40, CD40L, TNF-RII, IL-6Rα, B7-H3, ICAM-1, and FasL) were quantified by Luminex multiplex assay.

**Results and discussion:**

We observed significantly higher levels of EVs bearing PD-L1, CD40, TNF-RII and/or IL-6Rα in AIDS-NHL cases compared with controls. Using multivariate conditional logistic regression models adjusted for age and CD4^+^ T-cell count, we found that EVs bearing PD-L1 (OR = 1.93; 95% CI: 1.10 – 3.38), CD40 (OR = 1.97, 95% CI: 1.09 – 3.58), TNF-RII (OR = 5.06; 95% CI: 1.99 – 12.85) and/or IL-6Rα (OR = 4.67; 95% CI: 1.40 – 15.53) were significantly and positively associated with AIDS-NHL risk. In addition, EVs bearing these molecules were significantly and positively associated with non-CNS lymphoma: PD-L1 (OR = 1.94; 95% CI: 1.01 – 3.72); CD40 (OR = 2.66; 95% CI: 1.12 – 6.35); TNF-RII (OR = 9.64; 95% CI: 2.52 – 36.86); IL-6Rα (OR = 8.34; 95% CI: 1.73 – 40.15). These findings suggest that EVs bearing PD-L1, CD40, TNF-RII and/or IL-6Rα could serve as biomarkers for the early detection of NHL in PLWH.

## 1 Introduction

The incidence of B-cell non-Hodgkin lymphoma (NHL) is increased in chronic HIV infection. Even though the use of combination anti-retroviral therapy (cART) has improved the overall survival of people living with HIV (PLWH), AIDS-associated non-Hodgkin lymphoma (AIDS-NHL) remains a significant burden and cause of morbidity and mortality among PLWH in the post cART era (1–3). AIDS-NHL is classified as a high grade, extranodal disease and the most common tumor subtype is diffuse large B-cell lymphoma (DLBCL), followed by Burkitt lymphoma (BL) (2, 4–6). Other tumor subtypes are primary central nervous system lymphoma (PCNSL), plasmablastic lymphoma (PBL), and primary effusion lymphoma (PEL), which is seen less frequently (2, 4–6). Individuals with chronic HIV infection may develop lymphomas that are heterogenous in nature due to different pathogenic mechanisms that include chronic exposure to antigen, genetic mutations, dysregulation and production of pro-inflammatory cytokines, and the loss of immunoregulation of co-infections with oncogenic viruses [i.e., Epstein-Barr Virus (EBV), Kaposi’s sarcoma-associated herpesvirus (KSHV) or Human Herpesvirus 8 (HHV-8)] (1, 4, 5, 7). HIV infection may directly drive lymphomagenesis, which can be dictated by HIV viremia, the depth of CD4 nadir, the immunosuppressive state of the individual, and/or delayed ART treatment or interruptions in treatment (1, 2, 5, 7–10).

Extracellular vesicles (EVs) are secreted by all cells and can mediate cell to cell communication at distant sites. EVs are membrane-bound vesicles that can carry nucleic acids, proteins, lipids, and metabolites (11, 12). EVs also carry cargo that reflects its cell or tissue origin and cellular state (13–16). EVs carrying immunoregulatory molecules released by tumor cells can support cancer growth, metastasis, and resistance to chemotherapy (17). For example, EVs bearing bioactive programmed death-ligand 1 (PD-L1), can systematically counter anti-tumor immunity and evade immune surveillance (18). PD-L1 is one of the principal ligands of programmed cell death protein 1 (PD-1), which can be expressed on myeloid and lymphoid cells, epithelial cells, and tumor cells (19).

EVs play a role in HIV viral pathogenesis by delivering virus-associated membrane proteins and microRNAs to recipient cells, which can activate resting CD4^+^ T-cells to replicate HIV, and thus, induce the activation of latent reservoirs (20–22). EVs that are secreted by HIV-infected cells can also promote inflammation (23–25), induce apoptosis of nearby CD4^+^ T-cells (26), augment KSHV infectivity (27), and promote the growth and progression of cancer (28–30). EVs may also contribute to the development of NHL by promoting immune, cancer, and tissue-resident cell to cell crosstalk in the tumor microenvironment (31). Moreover, EVs found in peripheral blood circulation may serve as important biomarkers for hematological malignancies as early detection markers and measures of risk and disease progression (32–34). In a previous study, we found that AIDS-NHL patients had elevated levels of plasma derived EVs bearing PD-L1, CD40, CD40L and/or TNF-RII, when measured at the pre-treatment visit in the AIDS Malignancy Consortium (AMC) 034 (AMC-034) clinical trial (rituximab plus concurrent infusional EPOCH chemotherapy) (35). We also found that EVs bearing PD-L1 significantly and positively correlated with plasma levels of IL-10 before cancer treatment (35). Moreover, EVs bearing CD40, CD40L and/or TNF-RII significantly and positively correlated with baseline plasma levels of molecules shown to be elevated years prior to an AIDS-NHL diagnosis, such as IL-10, CXCL13, sCD25, sTNF-RII, and IL-18 (35). In this study, we sought to determine associations between EVs bearing PD-L1 and other molecules important for B-cell activation and/or differentiation with AIDS-NHL risk.

## 2 Materials and methods

### 2.1 Study participants

Subjects were participants in the Los Angeles Clinical Research Site (CSR) of the MACS/WIHS Combined Cohort Study (MWCCS), which has been previously described (36, 37). Archived serum samples were used for this study and were specifically from the Multicenter AIDS Cohort Study (MACS), now part of the MWCCS. The MACS was a longitudinal study of HIV-1 infection among gay and bisexual men in the United States that began in 1984 and is ongoing (36, 37). Archived serum samples were selected from PLWH who went on to develop AIDS-NHL (cases, N = 51), as well as from controls (N = 52) who were matched to cases on HIV status, study visit date (within 2 years), age (within 10 years), and CD4^+^ T-cell count (within 100 cells).

### 2.2 Ethics approval statement

The studies involving human participants (MWCCS) were reviewed and approved by UCLA human subjects research review committee (IRB# 20-002292). Written informed consent to participate in this study was provided by the participants. All specimens and any clinical information provided by the MWCCS were de-identified.

### 2.3 Isolation of EVs from serum samples

Serum samples (0.5 ml volume) were centrifuged at 2,000 x g for 30 minutes at room temperature to remove cell debris. The supernatant of clarified serum was then mixed with 0.2 volumes of total exosome isolation reagent as per manufacturer’s instructions (Catalog # 4478360; Invitrogen by Thermo Fisher Scientific, Waltham, MA, USA). The samples were incubated for 30 minutes at 4°C and then centrifuged at 10,000 x g for 10 minutes at room temperature to pellet the EVs, and the supernatant was discarded. EV pellets were resolubilized with 300 μl of 1X PBS and then stored at 4°C or at −20°C for downstream analyses.

### 2.4 Characterization of serum derived EVs by western blot

EV protein was quantified by Micro BCA assay using a 96-well plate format (Catalog # 23235; Micro BCA Protein Assay Kit, Thermo Fisher Scientific). Plates were read at an absorbance of 562 nm using a VersaMax Absorbance Microplate Reader (Molecular Devices, LCC., San Jose, CA, USA), and data was analyzed via the SoftMax Pro software (5.4). EV samples were prepared for western blot to measure the expression of exosomal markers (CD9 and TSG101) and a non-exosomal marker, Calnexin or CANX, as previously described (35). Blots were probed with primary antibodies: mouse anti-human CD9 (monoclonal in supernatant) (Catalog # 602.29 cl.11; Developmental Studies Hybridoma Bank (DSHB), Iowa City, IA, USA) at 1:50; rabbit anti-human TSG101 (Catalog # 28283-1-AP; Proteintech, Rosemont, IL, USA) at 1:1,000; and mouse anti-human CANX (Calnexin monoclonal antibody (AF18), Invitrogen, Catalog # MA3027; Fisher Scientific, USA) at 1:500. Blots were then probed with infrared (IR) fluorescently labeled secondary antibodies (IRDye, LI-COR, Lincoln, NE, USA), as previously described (35). Briefly, goat anti-mouse secondary antibody (IRDye 800CW) was used for detection of CD9 or CANX (1:10,000), and goat anti-rabbit secondary antibody (IRDye 680RD) was used for detection of TSG101 (1:10,000). Blots were scanned at 700 nm or 800 nm using a ChemiDoc Touch Imaging System (Bio-Rad; Hercules, CA, USA) at the UCLA AIDS Institute.

### 2.5 Quantification of serum derived EVs bearing different cell surface markers by Luminex-based multiplex immunometric assay

EV samples (60 μl) were treated with 1X PBS containing 0.1% Tween 20 to inactivate HIV. Samples were prepared in duplicate and loaded onto customized plates for Luminex-based multiplex immunometric assay. Customized plates were pre-coated with analyte-specific antibodies against PD-L1, CD40, CD40L, TNF-RII, CD126 (IL-6Rα), CD276 (B7-H3), CD54 (ICAM-1), and Fas Ligand (FasL) as provided by R&D Systems (Minneapolis, MN, USA), and as previously described (35). Standard curves were generated for each molecule. The average standard curve values for PD-L1 ranged from 1.84 to 1369.69 pg/ml; CD40 range: 6.63 to 4,749.92 pg/ml; CD40L range: 56.05 to 39,961.99 pg/ml; TNF-RII range: 3.20 to 2,359.99 pg/ml; IL-6Rα range: 69.98 to 51,044.27 pg/ml; B7-H3 range: 307.10 to 23,485.12 pg/ml; ICAM-1 range: 2,146.40 to 1,369,567.86 pg/ml; and FasL range; 3.04 to 2,169.62 pg/ml. In some cases, values needed to be extrapolated using fluorescence signals above the background level of detectable fluorescence, but below the lowest value of the standard curve, as previously described (35). Fluorescence intensity was quantified using a BioPlex 200 (Luminex) System Analyzer (Bio-Rad, Hercules, CA, USA). The data was analyzed using the BioPlex Manager (v 4.1.1) software program.

### 2.6 Measuring CD20 concentration on EVs by ELISA

CD20 was measured on the surface of serum derived EVs using 96-well ELISA plates coated with Human B-lymphocyte MS4A1 (CD20) (Catalog # MBS283766; MyBioSource, Inc. San Diego, CA, USA). Briefly, EV samples were diluted at 1:2 in 1X PBS containing 0.1% Tween 20 before loading onto ELISA plates and the assay was carried out according to manufacturer’s instructions (MyBioSource, Inc.). Results were acquired for CD20 standards and EV samples from two independent reproducible readings of duplicate wells at 450 nm using a VersaMax Absorbance Microplate Reader (Molecular Devices, LCC., San Jose, CA, USA) with SoftMax Pro Software (5.4) for Data Acquisition & Analysis.

### 2.7 Statistical analysis

Each biomarker was natural log-transformed to come near a normal distribution of the values in the study population. Data below the lower limit of detection (LLD) was substituted with a value equal to half of the LLD value acquired for any given biomarker, and specifically when <10% of the values had undetectable signals. Odds ratios (ORs) and 95% confidence intervals (CI) were determined using multivariate conditional (fixed-effects) logistic regression models adjusted for age and CD4^+^ T-cell count. A matched case-control design was implemented in the regression models by adding a matched set term. For most of the biomarkers (PD-L1, CD40, TNF-RII, IL-6Rα, CD40L, and CD20), OR values represent one unit increase in natural log-transformed values. For B7-H3, ICAM-1, and FasL, more than 10% of values were below the LLD, thus OR values are presented as detectable versus undetectable. Because of the varying degree of prior evidence of association among the biomarkers investigated in this study, and expected level of correlation between closely related markers, there was no obvious method to correct for multiple testing. However, to prioritize our results for interpretation and future studies, associations with *p* values less than or equal to 0.01 were considered significant, whereas associations with *p* values less than 0.05 were considered nominally significant.

## 3 Results

### 3.1 Study participant characteristics

All study participants were male, and most were white, non-Hispanic (**Table 1**). Serum samples from cases were selected from visits prior to the diagnosis of AIDS-NHL with a mean of 1.25 years (range of 2 to 36 months) pre-AIDS-NHL diagnosis. The median age of cases and controls were similar (42 and 41 years, respectively) (**Table 1**). The median CD4^+^ T-cell count was lower in cases compared to controls (165 versus 243 cells/mm^3^). Among HIV+ pre-AIDS-NHL cases, 55% were of diffuse large B-cell lymphoma (DLBCL) subtype, 22% of Burkitt lymphoma (BL) subtype, and 23% had a not otherwise specified (NOS) lymphoma (**Table 1**). **Table 1** also summarizes the number (and percent) of HIV+ pre-AIDS-NHL cases classified as non-CNS (84%) and CNS (16%) lymphoma.

**Table 1:** Characteristics of MWCCS participants.

	Controls	pre-AIDS-NHL cases
**N**	52	51
**Age, median ± SD**	41 ± 9	42 ± 9
**Race/ethnicity (N, %)** White, non-Hispanic White, Hispanic Black, non-Hispanic	51 (98%) 1 (2%) 0 (0%)	45 (88%) 5 (10%) 1 (2%)
**CD4^+^ T-cells (cells/mm^3^), median ± SD**	243 ± 258	165 ± 257
AIDS-NHL subtype		
DLBCL^1^	N/A^4^	28 (55%)
BL^2^	N/A	11 (22%)
NOS^3^	N/A	12 (23%)
CNS^5^	N/A	8 (16%)
Non-CNS	N/A	43 (84%)

^1^DLBCL, diffuse large B-cell lymphoma.

^2^BL, Burkitt lymphoma.

^3^NOS, not otherwise specified (NOS) lymphoma.

^4^N/A, not applicable.

^5^CNS, Central Nervous System.

### 3.2 EVs isolated from serum of HIV+ controls and HIV+ pre-AIDS-NHL cases express EV specific markers

**Figure 1:**
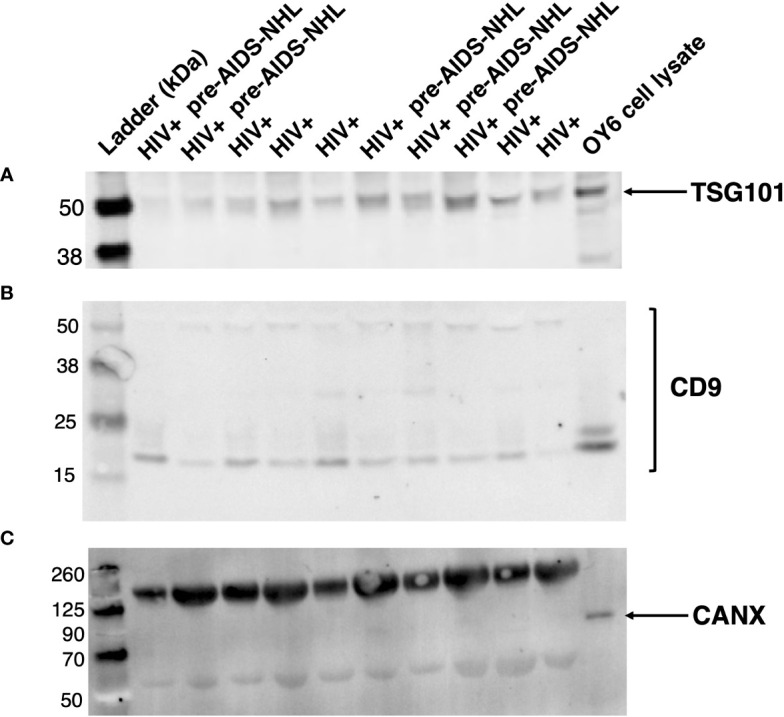
EVs isolated from serum of HIV+ and HIV+ pre-AIDS-NHL samples express EV-specific markers. Western blots of EVs isolated from serum of HIV+ controls and HIV+ pre-AIDS-NHL cases demonstrate the presence of EV specific markers **(A)** TSG101 and **(B)** CD9, and the absence of **(C)** Calnexin or CANX (ER chaperone and non-EV marker) on EVs isolated from serum samples. The expected molecular weight of TSG101 is ~44 kDa. The antigen molecular weight of CD9 is 25.4 kDa but CD9 protein can be observed as two species migrating near 25 kDa and 27 kDa, and multiple bands have also been observed at higher molecular weights. Whole cell lysate from OY6 (B-cell lymphoblastoid cell line (LCL)) was used as a positive control for CANX protein expression. The expected molecular weight of CANX is ~67 kDa but it may run as an ~90 kDa protein on SDS-PAGE. Note, the bands shown in the blot for CANX above 125 kDa and near 50 kDa pertain to IgG antibody complex bands that appear in serum samples, which are recognized by the secondary goat anti-mouse IgG (H+L) antibody (LI-COR IRDye 800CW) used to visualize the mouse anti-human CANX primary antibody. Secondary antibody complex bands were not detected in the OY6 cell lysate. 42 μg of total EV protein was loaded into each well. Blots for TSG101, CD9, and CANX were imaged for 30, 60, and 30 seconds, respectively. Results are representative of one sample set of EVs isolated from HIV+ controls (N = 5) and HIV+ pre-AIDS-NHL (N = 5) cases matched by age, visit, and CD4^+^ T-cell count.

EVs were isolated from archived serum using precipitation and centrifugation methods, as previously described (35). EVs isolated from serum samples of HIV+ controls and HIV+ pre-AIDS-NHL cohort participants were characterized by quantifying the expression of EV specific markers, CD9 and TSG101, to verify their identity as EVs. Whole cell lysate from OY6, a B-cell lymphoblastoid cell line (LCL), was included as a positive control for Calnexin or CANX, a lectin chaperone found in the ER and that is commonly used as a non-EV marker. CD9, TSG101, and CANX protein expression was assessed by Western blot. All serum derived EVs expressed CD9 and TSG101 (**Figures 1A, B**). CANX was detected in the OY6 cell lysate and not in serum derived EVs (**Figure 1C**).

### 3.3 Serum derived EVs bearing PD-L1, CD40, TNF-RII and/or IL-6Rα are significantly associated with AIDS-NHL risk

We then measured the levels of immunoregulatory molecules PD-L1 and B7-H3 on EVs, and molecules associated with B-cell activation and differentiation (CD40, CD40L, TNF-RII, IL-6Rα, ICAM-1), and FasL by Luminex-based multiplex immunometric assay. CD20 expression on EVs was determined by ELISA. Individual values for each biomarker and for each participant group [HIV+ controls (N=52) and HIV+ pre-AIDS-NHL (N=51)] are provided in **Supplementary Figure 1**. Data was natural log-transformed to derive normal distribution values for each biomarker (**Table 2**). When adjusting for age and CD4^+^ T-cell count in a multivariate conditional logistic regression model, elevated levels of EVs bearing PD-L1, CD40, TNF-RII and/or IL-6Rα were strongly and positively associated with increased odds of AIDS-NHL risk: PD-L1 (OR = 1.93; 95% CI: 1.10 – 3.38; *p* = 0.022), CD40 (OR = 1.97, 95% CI: 1.09 – 3.58; *p* = 0.025), TNF-RII (OR = 5.06; 95% CI: 1.99 – 12.85; *p* = 0.001) and/or IL-6Rα (OR = 4.67; 95% CI: 1.40 – 15.53; *p* = 0.012) (**Table 3**).

**Table 2:** Summary of mean natural log (ln)-transformed values for each biomarker.

EV Biomarker	Controls	pre-AIDS-NHL cases
	Mean value of ln transformed biomarker levels (N = 52)	Mean value of ln transformed biomarker levels (N = 51)
PD-L1	1.04	1.77
CD40	2.70	3.16
TNF-RII	4.45	4.99
IL-6Rα	8.88	9.10
B7-H3^1^	64.20	62.48
CD40L	6.05	6.22
ICAM-1^1^	79.30	86.30
FasL^1^	35.90	54.90
CD20	2.11	2.16

^1^For B7-H3, ICAM-1, and FasL, more than 10% of values were below the lower limit of detection, thus values are presented as percent detectable values.

**Table 3:** Results of the adjusted multivariate conditional logistic regression model for EV biomarkers and AIDS-NHL risk.

EV Biomarker	Controls (N)	pre-AIDS-NHL cases (N)	Odds Ratio (OR)^1,2^	95% CI^2^	*p*-value^2^
PD-L1	52	51	1.93	1.10 – 3.38	0.022
CD40	52	51	1.97	1.09 – 3.58	0.025
TNF-RII	52	51	5.06	1.99 – 12.85	0.001
IL-6Rα	52	51	4.67	1.40 – 15.53	0.012
B7-H3	52	51	0.29	0.03 – 2.94	0.294
CD40L	52	51	1.26	0.75 – 2.10	0.379
ICAM-1	52	51	1.73	0.41 – 7.26	0.453
FasL	52	51	7.52	0.94 – 60.45	0.058
CD20	52	51	1.65	0.74 – 3.68	0.218

^1^Odds Ratio (OR) is a measure of one unit increase on the natural logarithm (ln) scale for each biomarker, except for B7-H3, ICAM-1, and FasL, where more than 10% of their values fell below the lower limit of detection. Therefore, OR values are presented as detectable versus undetectable values.

^2^OR, 95% CI, and p-values are shown for each biomarker as determined by a multivariate conditional logistic regression model adjusted by age and CD4^+^ T-cell count.

In stratified analysis by tumor subtype, we did not determine any significant associations between EVs bearing PD-L1, CD40, FasL, IL-6Rα, B7-H3, CD40L, ICAM-1 or CD20 with DLBCL, BL, and NOS lymphoma subtypes. However, we did find a meaningful association between elevated levels of EVs bearing TNF-RII with increased odds of AIDS-NHL risk and DLBCL tumor subtype (OR = 7.90; 95% CI: 1.74 – 35.92; *p* = 0.008).

### 3.4 EVs bearing PD-L1, CD40, TNF-RII and/or IL-6Rα are associated with AIDS-NHL risk and non-CNS lymphoma

In stratified analysis by CNS and non-CNS lymphoma type, we determined significant associations between EVs bearing PD-L1 (OR = 1.94; 95% CI: 1.01 – 3.72, *p* = 0.046), CD40 (OR = 2.66; 95% CI: 1.12 – 6.35; *p* = 0.027), TNF-RII (OR = 9.64; 95% CI: 2.52 – 36.86; *p* = 0.001) and/or IL-6Rα (OR = 8.34; 95% CI: 1.73 – 40.15; *p* = 0.008) and non-CNS lymphoma (**Table 4**). We did not observe any significant associations with CNS lymphoma (**Table 4**).

**Table 4:** Results of the adjusted multivariate conditional logistic regression model for non-CNS and CNS lymphoma.

EV Biomarker	non-CNS (systemic lymphoma) (N = 43)			CNS lymphoma (N = 8)		
	Odds Ratio (OR)^1^	95% CI^1^	*p*-value^1^	Odds Ratio (OR)	95% CI	*p*-value
PD-L1	1.94	1.01 – 3.72	0.046	2.72	0.28 – 26.19	0.386
CD40	2.66	1.12 – 6.35	0.027	1.70	0.44 – 6.53	0.440
TNF-RII	9.64	2.52 – 36.86	0.001	1.74	0.45 – 6.71	0.418
IL-6Rα	8.34	1.73 – 40.15	0.008	2.16	0.23 – 20.51	0.504
B7-H3	0.26	0.02 – 2.81	0.265	NA^2^	NA	NA
CD40L	1.21	0.67 – 2.19	0.532	0.62	0.12 – 3.22	0.570
ICAM-1	2.18	0.39 – 12.11	0.373	18.01	0.09 – 3554.58	0.284
FasL	3.99	0.46 – 34.68	0.210	NA	NA	NA
CD20	2.12	0.84 – 5.39	0.113	0.45	0.02 – 11.94	0.636

^1^OR, 95% CI, and p-values are shown for each biomarker as determined by a multivariate conditional logistic regression model adjusted by age and CD4^+^ T-cell count.

**^2^** Not available, NA: OR and 95% CI values are not available for FasL and B7-H3 in CNS lymphoma because of no within group variance.

## 4 Discussion

In this study, we measured serum levels of EVs bearing different cell surface markers in HIV+ and HIV+ pre-AIDS-NHL participants of the MWCCS. Elevated levels of EVs bearing PD-L1, CD40, TNF-RII and/or IL-6Rα were observed in serum of HIV+ pre-AIDS-NHL cases compared to HIV+ controls. Others have found that PD-L1, TNF-RII, and IL-6 could serve as biomarkers of NHL (38–40). We also found significant associations between increased levels of EVs bearing PD-L1, CD40, TNF-RII and/or IL-6Rα with AIDS-NHL risk and more specifically with the development of non-CNS AIDS-NHLs. Moreover, no significant associations were found between EVs expressing these molecules with DLBCL and BL tumor subtypes. However, we did find a meaningful association between elevated levels of EVs bearing TNF-RII with increased risk for the development of AIDS-NHL and DLBCL. It is possible that the lack of significant associations between the biomarkers examined, and BL or NOS lymphoma, is due to the small number of cases for this subtype.

Pre-tumor cells can secrete EVs that contain unique molecules that promote the primary tumor growth or that mediate interactions with immune or tissue resident cells. Tumor cells can also release EVs bearing PD-L1 on their surface to promote immunosuppression and can give rise to pre-metastatic niches (41–45). In prior work, we found that individuals infected with HIV had elevated numbers of PD-L1^+^ B-cells, B regulatory (Breg) cells, and PD-L1 expressing Bregs in circulation at 1 to 4 years prior to an AIDS-NHL diagnosis when compared with HIV+ individuals without an AIDS-NHL diagnosis (46). Thus, it is plausible that the elevated levels of EVs bearing PD-L1 in serum from pre-AIDS-NHL cases in this study could be derived from PD-L1 expressing B-cells or Bregs, which could be a response to systemically suppress the immune system to inhibit HIV-infected CD4^+^ T-cells.

Activated B-cells expressing CD40 function as professional antigen-presenting cells (APCs) that can interact with T-cells following ligation to its ligand, CD40 ligand (CD40L or CD154) (47). CD40 signaling leads to canonical and non-canonical pathways of NF-κB signaling (48, 49). Deregulated expression of CD40 on tumor cells may contribute to lymphomagenesis *in vivo* by mediating non-canonical signaling, prolonged survival, and increased proliferation (50). The expression of CD40 on malignant B-cells can serve as a prognostic factor of DLBCL (51). In this work, we observed elevated levels of CD40 bearing EVs in circulation of pre-AIDS-NHL subjects, which can engage and signal activated T-cells and/or facilitate their recruitment to distant sites.

The tumor necrosis factor (TNF) cytokine is a driver of inflammation and has been implicated in the development of cancer (52). In the context of cancer, TNF binds its receptors TNF-RI and TNF-RII to promote cell proliferation (53). More specifically, TNF-RII is overexpressed in malignant cells to promote cell proliferation in many different types of cancers, including malignant B-cell lymphomas (54, 55). Thus, EVs secreted by cancer cells overexpressing TNF-RII could deliver TNF-RII to recipient cells. We have previously shown that serum levels of TNFα are significantly elevated in HIV+ individuals part of the MWCCS, and these elevated levels are strongly associated with increased risk of AIDS-NHL (10). Thus, in this study we were interested in evaluating an association between TNF-RII expressing EVs and AIDS-NHL risk. Our findings support this hypothesis and highlight a potential role of TNF-RII expressing EVs in lymphomagenesis, in which TNF-RII may be potentially delivered to recipient cells via EVs to induce TNF-RII-mediated signaling, including autocrine signaling events, and to promote cell proliferation and/or migration.

IL-6 is a cytokine with pleiotropic activities. IL-6Rα is a receptor for IL-6 that exists in soluble and transmembrane forms (56). IL-6 that binds soluble IL-6Rα leads to pro-inflammatory responses, while IL-6 that binds the transmembrane form of IL-6Rα leads to anti-inflammatory messages (56). When IL-6 binds the soluble or membrane-bound form of IL-6Rα it causes the formation of a complex that is recognized by the IL-6 receptor subunit-β (gp130) further inducing the activation of the JAK/STAT3 signaling pathway, which can lead to cell differentiation and proliferation, migration, and metabolic changes (56). Dysregulated IL-6 signaling can contribute to the progression of pathological conditions, including cancer (56). In this study, we were interested in measuring IL-6Rα in EVs as IL-6 levels are significantly elevated in HIV-infected individuals years prior to an AIDS-NHL diagnosis (2, 7, 57). The elevated levels of EVs bearing IL-6Rα in pre-AIDS-NHL cases may bind cell membranes through receptor-ligand interactions and induce signaling in recipient cells. Studies have shown that EVs isolated from human serum carry the full-length version of IL-6Rα (CD126) (58). Others have shown that EVs that carry and transport the full-length and membrane-bound version of IL-6Rα (CD126) are able to induce STAT3 phosphorylation via gp130 in recipient cells (59).

Our findings collectively suggest that increased levels of EVs bearing molecules of B-cell immune activation, such as CD40, TNF-RII, IL-6Rα, and immunomodulatory molecules (PD-L1) may affect B-cell lymphomagenesis years prior to an AIDS-NHL diagnosis and thus, could help identify key molecules for diagnosis, immune escape, and other molecular factors associated with lymphoma initiation/development. Further assessing correlations between EVs and soluble PD-L1 (or soluble forms of CD40, TNF-RII, and IL-6Rα), immunomodulatory cytokines/chemokines, and/or circulating immune cells may elucidate additional factors driving the development of AIDS-NHL.

EVs secreted into peripheral blood circulation can carry bioactive molecules from different immune cells, such as T-cells, NK cells, monocytes, macrophages, and dendritic cells, including EVs shed by cells from the tissue microenvironment (i.e., stromal cells, fibroblasts, endothelial cells). Our study specifically investigated molecules associated with chronic B-cell activation/differentiation during HIV infection, including B7 family of immune regulatory ligands. Therefore, expanding these studies to investigate the role of EVs bearing molecules from other immune cell types, including EVs from tissue resident cells would be important in further understanding lymphomagenesis.

### 4.1 Limitations of this study

The serum samples used for this study were originally from the MACS, a longitudinal study of HIV infection among gay and bisexual men that began in 1984. Therefore, our study was limited by not having access to samples from female participants infected with HIV and that went on to develop AIDS-NHL. Thus, the inclusion of male, female, and transgender study participants and from different racial and ethnic minority groups is significantly important, including the inclusion of study samples from Black/African American and Hispanic/Latino communities, which are representative of the population disproportionately affected by HIV in the United States (60) and in Los Angeles County (61). A limitation of this study is the small sample size of study participants, which limits power and additional analysis by tumor subtype. HIV viral load data were unavailable for most cohort participants and evaluating a relationship between EVs and other clinical features, such as HIV viral load, stage of disease and/or tumor subtype is not possible. Moreover, we were limited in having one pre-diagnosis sample from each HIV+ participant that went on to develop AIDS-NHL. The majority of HIV+ participants from this group died shortly after an NHL diagnosis and very few provided a post-diagnosis sample. Therefore, we were unable to study the potential role of EVs after an AIDS-NHL diagnosis and/or evaluate their prognostic value.

Our study investigated molecules for which we had preliminary evidence in AMC-034 clinical trial participants (35), and for which robust immunoassays were available. However, assessment of other immunoregulatory molecules carried by EVs, and their variable surface composition, will provide insight into the emerging role of EVs in modulating the immune response during different stages of lymphomagenesis, and provide insight on how EVs contribute to the highly heterogenous nature of tumors. Our study is also limited in not knowing the immune cell origin of EVs released into peripheral blood circulation. Multiplex proximity extension assays and proteomic profiling of body-fluid derived EVs with matched cell lysates from human tumor biopsies or tumor derived cell lines could help trace EVs to their parental cells and further allow the characterization of cell type-specific signatures in highly heterogeneous tissue and/or permit the identification of biomarkers important for predicting tumor progression and metastasis (62–65).

### 4.2 Conclusions

The results described in this study show that EVs bearing PD-L1, CD40, TNF-RII and/or IL-6Rα are significantly associated with AIDS-NHL risk and thus, may serve as biomarkers of AIDS-NHL. EVs have the potential to facilitate communication between malignant B-cells and other immune cells found in the tumor microenvironment (66). The expression of PD-L1, CD40, TNF-RII and/or IL-6Rα on the surface of EVs could modulate immune responses and play are role in early events of lymphomagenesis. PD-L1-expressing EVs can inhibit T-cells found in draining lymph nodes and/or at sites distant from the cancer cell origin (67, 68), and can suppress anti-tumor immunity to promote the progression of cancer (41, 43, 45, 69, 70). Thus, investigating whether EVs bearing PD-L1, and/or other B7-molecules, have immunosuppressive properties that may inhibit T-cell activation and/or anti-tumor responses in AIDS-NHL is of importance. Future studies will determine if EVs that promote tumor progression are associated with drivers of inflammation and microbial translocation, previously shown to be elevated before an AIDS-NHL diagnosis (8, 10, 57, 71). Moreover, characterizing the host proteome (i.e., membrane bound proteins, cytosolic proteins, and molecules associated with antigen presentation), DNA and RNA content (i.e., biologically active miRNAs and lncRNAs), and HIV-derived cargo of EVs will provide insight into the pre-tumor material delivered to recipient cells, as well as identify key molecules involved in B-cell immune dysfunction and/or oncogenic transformation, which may be important for sustaining tumor growth, metastasis and/or remodeling the tumor microenvironment.

## Data availability statement

The raw data supporting the conclusions of this article will be made available by the authors, without undue reservation.

## Ethics statement

The studies involving human participants (MWCCS) were reviewed and approved by UCLA human subjects research review committee (IRB# 20-002292). Written informed consent to participate in this study was provided by the participants. All specimens and any clinical information provided by the MWCCS were de-identified.

## Author contributions

LEM: Investigation, Writing – original draft, Writing – review & editing, Data curation, Methodology. LIM: Methodology, Writing – review & editing, Data curation, Validation. YG: Data curation, Methodology, Validation, Writing – review & editing. PH: Data curation, Methodology, Writing – review & editing. RD: Writing – review & editing, Funding acquisition, Resources, Supervision. SH: Resources, Supervision, Writing – review & editing, Formal Analysis, Writing – original draft. ME: Formal Analysis, Resources, Supervision, Writing – original draft, Writing – review & editing, Conceptualization, Funding acquisition, Investigation, Project administration, Visualization.
