# Supplementary material for: Extracellular vesicles as biomarkers for AIDS-associated non-Hodgkin lymphoma risk
Source: Front Immunol. 2023 Sep 22;14:1259007. doi: 10.3389/fimmu.2023.1259007 (PMC10556683; doi:10.3389/fimmu.2023.1259007)
Supplement: Supplementary file 1 [file DataSheet_1.docx]

Supplementary Material

# Extracellular vesicles as biomarkers for AIDS-associated non-Hodgkin lymphoma risk

# Laura E. Martínez^1,2^, Larry I. Magpantay^1,2^, Yu Guo^1,2^, Priya Hegde^1^, Roger Detels^3^, Shehnaz K. Hussain^4^, and Marta Epeldegui^1,2,5^

# ^1^UCLA AIDS Institute and David Geffen School of Medicine, University of California, Los Angeles, CA, United States

# ^2^Department of Obstetrics and Gynecology, David Geffen School of Medicine, University of California, Los Angeles, CA, United States

# ^3^Jonathan and Karin Fielding School of Public Health, University of California, Los Angeles, CA, United States

# ^4^Department of Public Health Sciences, School of Medicine and Comprehensive Cancer Center, University of California, Davis, CA, United States

# ^5^Jonsson Comprehensive Cancer Center, University of California, Los Angeles, CA, United States

# *Correspondence:

# Marta Epeldegui, Ph.D., University of California Los Angeles, AIDS Institute, Biomedical Sciences Research Building Room 173, Los Angeles, CA 90095. Phone: (310) 206-6846

# [mepeldegui@mednet.ucla.edu](mailto:mepeldegui@mednet.ucla.edu)

# Supplementary Figures

**
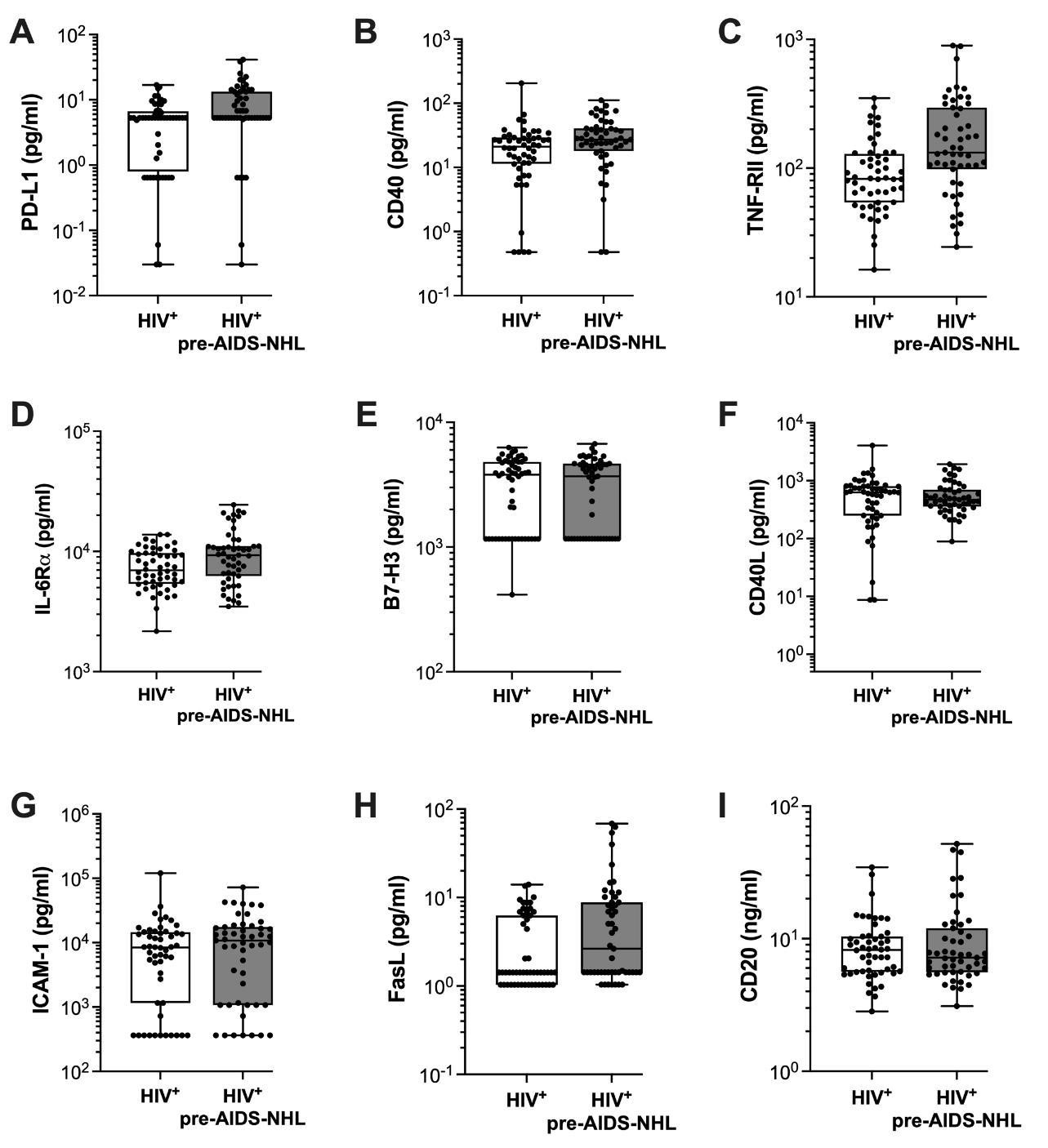
**

**Supplementary Figure 1. Biomarker levels of EVs bearing molecules of interest.** Biomarker levels of EVs bearing the molecules of interest for this study were measured by Luminex-based multiplex assay. Shown are box and whisker plots with continuous values for each biomarker, which were dichotomized for the regression model (see **Table 2**). Values below the lower limit of detection (LLD) were imputed as half of the LLD, thus, these values cluster as minimum values in each plot. Continuous values are shown within the box and whisker plots for **(A)** PD-L1, **(B)** CD40, **(C)** TNF-RII, **(D)** IL-6Rα, **(E)** B7-H3, **(F)** CD40L, **(G)** ICAM-1, **(H)** FasL, and **(I)** CD20 for HIV+ controls (N = 52) and HIV+ pre-AIDS-NHL cases (N = 51). Each individual point is superimposed on the box plot. The box represents the 95% limit of the mean value. The line inside the box indicates the mean value. Minimum and maximum values are provided as whiskers.
